# Supplementary figures and images for: Efficacy of pharmacological and non-pharmacological therapy on chronic cancer pain intensity of adults with cancer: A network meta-analysis protocol
Source: PLoS One. 2025 Jul 17;20(7):e0322651. doi: 10.1371/journal.pone.0322651 (PMC12270095; doi:10.1371/journal.pone.0322651)

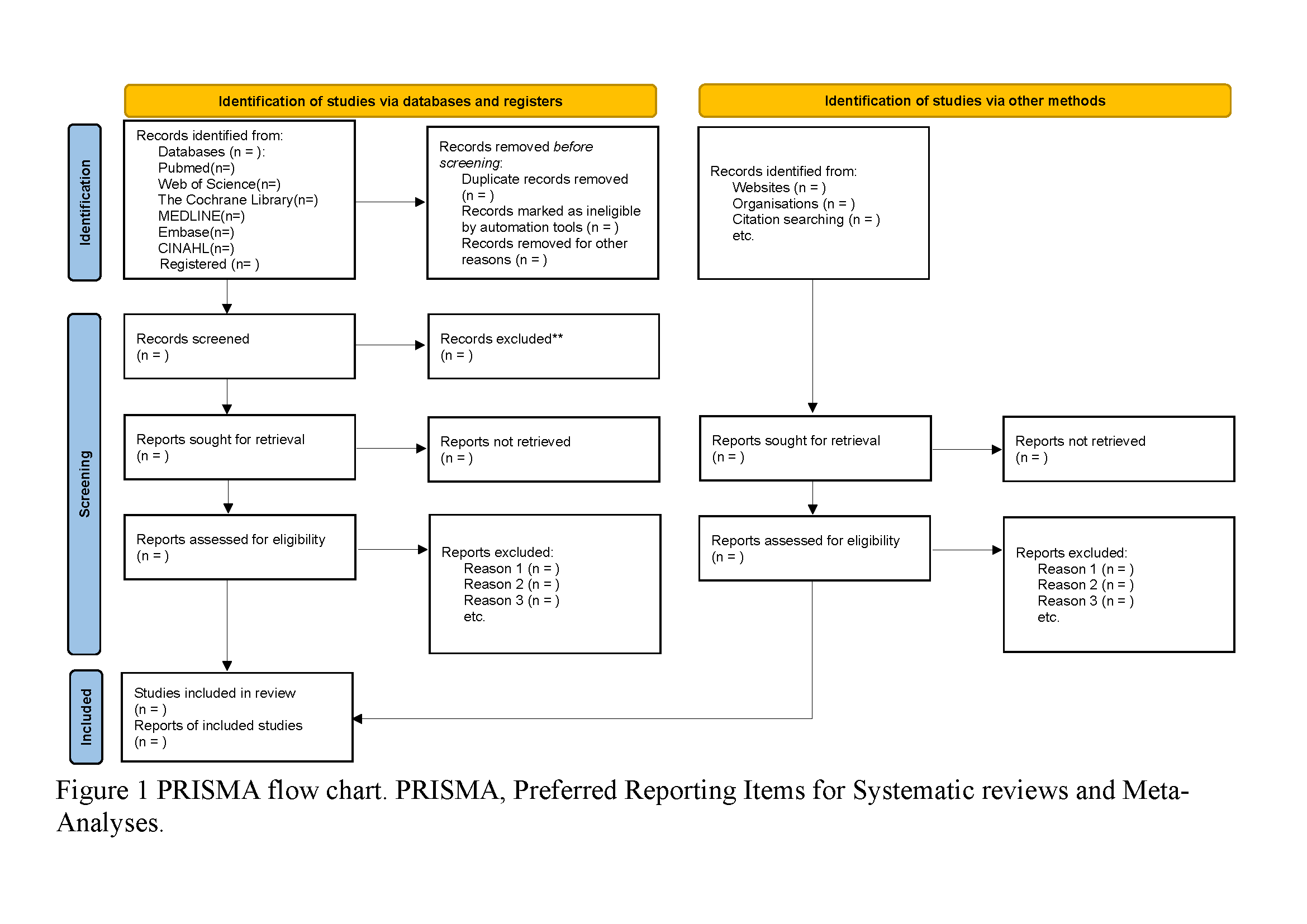

Supplement: S1 Fig — (TIF) [file pone.0322651.s001.tif]

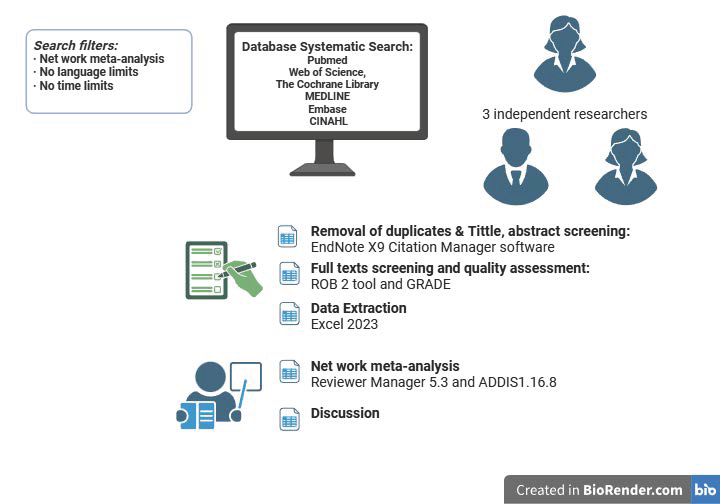

Supplement: S2 Fig — (TIF) [file pone.0322651.s002.tif]
