# Supplementary material for: Efficacy of pharmacological and non-pharmacological therapy on chronic cancer pain intensity of adults with cancer: A network meta-analysis protocol
Source: PLoS One. 2025 Jul 17;20(7):e0322651. doi: 10.1371/journal.pone.0322651 (PMC12270095; doi:10.1371/journal.pone.0322651)
Supplement: S3 File — (PDF) [file pone.0322651.s005.pdf]

S3 File Summary of PICOS eligibility criteria

	Inclusion criteria
Population	Adults with chronic cancer pain diagnosed with IASP criteria
Intervention	Non-pharmacological interventions
Comparison	pharmacological interventions, placebo, sham, no intervention
Outcome	Pain intensity, total effective rate of treatment, quality of life, adverse reaction
Study design	Randomized controlled trials (RCTs)
