# Supplementary material for: Efficacy of pharmacological and non-pharmacological therapy on chronic cancer pain intensity of adults with cancer: A network meta-analysis protocol
Source: PLoS One. 2025 Jul 17;20(7):e0322651. doi: 10.1371/journal.pone.0322651 (PMC12270095; doi:10.1371/journal.pone.0322651)
Supplement: S4 File — (PDF) [file pone.0322651.s006.pdf]

S3 File Definitions of each intervention node

Classes of therapies are identified based on interventions that are usually used to manage pain associated with cancer. Every intervention node will be examined regardless of its frequency, duration, intensity, or adverse reaction. The following definitions are derived from previously published systematic reviews and randomized controlled trials that examined the effectiveness of therapies for cancer pain:

1. Acupuncture

Acupuncture is referred to as "using traditional Chinese medicine theory to insert acupuncture into acupoints according to WHO standard (1), such as Ashi point, Zusanli, Sanyinjiao, etc. Electroacupuncture will not be considered. The WHO acupuncture point criteria do not include studies on acupuncture interventions that have not been included (2, 3).

2. Cognitive behavioral therapy, mindfulness and meditation therapies,

2.1. Cognitive behavioral therapy –It will be defined as aimed at alterating of negative ideas and behavioral patterns toward pain, improving mental health and promoting behavioral change, and developing strategies to alleviate pain. The following technologies will be discussed: customized cognitive behavioral treatment programs, operational therapy, self-management education programs, and standard cognitive behavioral therapy(4). Besides, mindfulness and meditation, psychological therapy, and spiritual intervention will be also considered as well.

2.2. Mindfulness and meditation - It will be defined as a way to rest attention on an object, thing, or thought, and cultivate the ability to be in the present moment, non-judgmentally, with sensations, thoughts, emotions, etc(5, 6).

2.3. Psychological therapy - It will be defined as techniques and methods to help individuals cope with emotional difficulties, improve mental health, and address personal challenges. Therapists provide a supportive environment to explore feelings, thoughts, and behaviors, aiming to promote positive changes and enhance overall well-being(7).

2.4. Spiritual intervention - It will be defined as techniques or methods in the incorporation of religious, faith-based, or spiritual practices into medical and psychological care to support and improve a patient's psychological, emotional, and spiritual well-being. This can include activities such as prayer, spiritual counseling, religious rituals, scripture reading, or other practices that provide spiritual support(8).

3. Electrotherapy

Electrotherapy will be defined as treatments that use electrical energy to stimulate nerves and muscles for therapeutic purposes, such as pain relief, muscle rehabilitation, or to promote healing. This may include: transcutaneous electric nerve stimulation (TENS), electroacupuncture, and galvanic current(9, 10).

4. Hypnosis

Hypnosis will be defined that it is often induced by a hypnotherapist using verbal cues and mental imagery to help patients make positive changes, manage pain, or address psychological issues(11).

5. Massage

Any manipulation of soft tissue carried out with the hands or with the use of additional tools will be defined as massage. Any region of the body could have received the massage (2).

6. Music therapy

Music therapy will be defined as a therapeutic approach that uses music to address physical, emotional, cognitive, and distraction of pain in individuals(12).

7. Telephone intervention

Telephone intervention will be defined as a form of therapy or support delivered via phone calls, aimed at providing immediate assistance, counseling, and psychological support to individuals in pain(13).

8. Yoga, Taichi and Qigong

Yoga, Tai Chi, and Qigong will be defined as treatments that involve gentle physical exercises and mindfulness practices to reduce pain, improve physical function, and enhance overall well-being(14).

9. Virtual reality

Virtual reality will be defined as treatment for cancer pain patients using immersive VR technology that includes a computer capable of real-time animation, controlled by a set of sensory input devices, a position tracker, and a head-mounted device for visual output to distract and manage pain, reducing discomfort and improving overall well-being(15, 16).

10. Complementary therapies

Complementary therapies will be defined as non-mainstream treatments used alongside conventional medical treatments to help treat cancer pain, improve health, and enhance well-being. These can include methods like journaling, hand massage, and imagery, herbal medicine, Magnetic field therapy, etc(17, 18).

11. WHO Analgesic Ladder

WHO Analgesic Ladder will be defined as using non-opioid(nonsteroidal anti-inflammatory drugs, NSAIDs) analgesics for mild pain, weak opioid (hydrocodone, codeine, tramadol) analgesics for moderate pain, and potent opioids(morphine, methadone, fentanyl, oxycodone, buprenorphine, tapentadol, hydromorphone, oxymorphone) for severe and persistent pain(19).

12. Other potential medicines

Other potential medicines will be defined as any medicine that shows effectiveness in alleviating cancer pain, such as marijuana.

REFERENCES

1. Organization WSGoIANWH. A proposed standard international acupuncture nomenclature : report of a WHO scientific group: World Health Organization; 1991.
2. Epstein AS, Liou KT, Romero SAD, Baser RE, Wong G, Xiao H, et al. Acupuncture vs Massage for Pain in Patients Living With Advanced Cancer: The IMPACT Randomized Clinical Trial. JAMA Netw Open. 2023;6(11):e2342482.
3. Paley CA, Johnson MI, Tashani OA, Bagnall AM. Acupuncture for cancer pain in adults. Cochrane Database Syst Rev. 2015;2015(10):Cd007753.
4. Ruano A, García-Torres F, Gálvez-Lara M, Moriana JA. Psychological and Non-Pharmacologic Treatments for Pain in Cancer Patients: A Systematic Review and Meta-Analysis. Journal of pain and symptom management. 2022;63(5):e505-e20.
5. Cillessen L, Johannsen M, Speckens AEM, Zachariae R. Mindfulness-based interventions for psychological and physical health outcomes in cancer patients and

survivors: A systematic review and meta-analysis of randomized controlled trials. Psychooncology. 2019;28(12):2257-69.
6. Behzadmehr R, Dastyar N, Moghadam MP, Abavisani M, Moradi M. Effect of complementary and alternative medicine interventions on cancer related pain among breast cancer patients: A systematic review. Complementary therapies in medicine. 2020;49:102318.
7. Eccleston C, Fisher E, Craig L, Duggan GB, Rosser BA, Keogh E. Psychological therapies (Internet-delivered) for the management of chronic pain in adults. Cochrane Database Syst Rev. 2014;2014(2):Cd010152.
8. Kruizinga R, Hartog ID, Jacobs M, Daams JG, Scherer-Rath M, Schilderman JB, et al. The effect of spiritual interventions addressing existential themes using a narrative approach on quality of life of cancer patients: a systematic review and meta-analysis. Psychooncology. 2016;25(3):253-65.
9. Mao JJ, Liou KT, Baser RE, Bao T, Panageas KS, Romero SAD, et al. Effectiveness of Electroacupuncture or Auricular Acupuncture vs Usual Care for Chronic Musculoskeletal Pain Among Cancer Survivors: The PEACE Randomized Clinical Trial. JAMA Oncol. 2021;7(5):720-7.
10. Hurlow A, Bennett MI, Robb KA, Johnson MI, Simpson KH, Oxberry SG. Transcutaneous electric nerve stimulation (TENS) for cancer pain in adults. Cochrane Database Syst Rev. 2012;2012(3):Cd006276.
11. Sine H, Achbani A, Filali K. The Effect of Hypnosis on the Intensity of Pain and Anxiety in Cancer Patients: A Systematic Review of Controlled Experimental Trials. Cancer Invest. 2022;40(3):235-53.
12. Yangöz Ş T, Özer Z. The effect of music intervention on patients with cancer-related pain: A systematic review and meta-analysis of randomized controlled trials. J Adv Nurs. 2019;75(12):3362-73.
13. Ream E, Hughes AE, Cox A, Skarparis K, Richardson A, Pedersen VH, et al. Telephone interventions for symptom management in adults with cancer. Cochrane Database Syst Rev. 2020;6(6):Cd007568.
14. Buffart LM, van Uffelen JG, Riphagen, II, Brug J, van Mechelen W, Brown WJ, et al. Physical and psychosocial benefits of yoga in cancer patients and survivors, a systematic review and meta-analysis of randomized controlled trials. BMC Cancer. 2012;12:559.
15. Chow H, Hon J, Chua W, Chuan A. Effect of Virtual Reality Therapy in Reducing Pain and Anxiety for Cancer-Related Medical Procedures: A Systematic Narrative Review. Journal of pain and symptom management. 2021;61(2):384-94.
16. Zeng Y, Zhang JE, Cheng ASK, Cheng H, Wefel JS. Meta-Analysis of the Efficacy of Virtual Reality-Based Interventions in Cancer-Related Symptom Management. Integrative cancer therapies. 2019;18:1534735419871108.
17. Snyder M, Wieland J. Complementary and alternative therapies: what is their place in the management of chronic pain? The Nursing clinics of North America. 2003;38(3):495-508.
18. Cassileth BR, Deng G. Complementary and alternative therapies for cancer. The oncologist. 2004;9(1):80-9.
19. Cascella AAAJMHM. WHO Analgesic Ladder: StatPearls Publishing; 2023.
