# Supplementary material for: Efficacy of pharmacological and non-pharmacological therapy on chronic cancer pain intensity of adults with cancer: A network meta-analysis protocol
Source: PLoS One. 2025 Jul 17;20(7):e0322651. doi: 10.1371/journal.pone.0322651 (PMC12270095; doi:10.1371/journal.pone.0322651)
Supplement: S5 File — (PDF) [file pone.0322651.s007.pdf]

S4 File Search Strategy

Take PubMed as an example, the following keywords and Medical Subject Headings (MESH) will be used for searching articles:

#1 ("Cancer Pain" [MH] OR Cancer Pains [TIAB] OR Pain*, Cancer [TIAB] OR Cancer-Related Pain* [TIAB] OR Cancer Related Pain [TIAB] OR Pain*, Cancer-Related [TIAB] OR Cancer-Associated Pain [TIAB] OR Cancer Associated Pain* [TIAB] OR Pain*, Cancer-Associated [TIAB] OR Neoplasm-Related Pain [TIAB] OR Neoplasm Related Pain* [TIAB] OR Pain*, Neoplasm-Related [TIAB] OR Neoplasm-Associated Pain [TIAB] OR Neoplasm Associated Pain* [TIAB] OR Pain*, Neoplasm-Associated [TIAB] OR Tumor-Related Pain [TIAB] OR Pain*, Tumor-Related [TIAB] OR Tumor Related Pain* [TIAB] OR Tumor-Associated Pain* [TIAB] OR Pain*, Tumor-Associated [TIAB] OR Tumor Associated Pain [TIAB])

#2 ("Acupuncture" [MH] OR "Acupuncture Points" [MH] OR "Acupuncture Therapy" [MH] OR Electroacupuncture[MH] OR "Electric Stimulation Therapy" [MH] OR "Acupuncture, Ear" [MH] OR Auriculotherapy [MH] OR Acupuncture [TIAB] OR Acupuncture Points [TIAB] OR Acupuncture Therapy [TIAB] OR Electroacupuncture[TIAB] OR Electric Stimulation Therapy [TIAB] OR Acupuncture, Ear [TIAB] OR Auriculotherapy [TIAB] OR Pharmacoacupuncture [TIAB] OR Acupuncture Treatment* [TIAB] OR Treatment, Acupuncture [TIAB] OR Therapy, Acupuncture [TIAB] OR Pharmacoacupuncture Treatment [TIAB] OR Treatment, Pharmacoacupuncture [TIAB] OR Pharmacoacupuncture Therapy [TIAB] OR Therapy, Pharmacoacupuncture [TIAB] OR Acupotom*[TIAB] OR Acupuncture Point [TIAB] OR Point, Acupuncture [TIAB] OR Points, Acupuncture [TIAB] OR Acupoints [TIAB] OR Acupoint [TIAB] OR Therapy, Electric Stimulation [TIAB] OR Stimulation Therapy, Electric [TIAB] OR Electrotherapy [TIAB] OR Therapeutic Electric Stimulation

[TIAB] OR Electric Stimulation, Therapeutic [TIAB] OR Stimulation, Therapeutic Electric [TIAB] OR Electrical Stimulation Therapy [TIAB] OR Stimulation Therapy, Electrical [TIAB] OR Therapy, Electrical Stimulation [TIAB] OR Therapeutic Electrical Stimulation [TIAB] OR Electrical Stimulation, Therapeutic [TIAB] OR Stimulation, Therapeutic Electrical [TIAB] OR Interferential Current Electrotherapy [TIAB] OR Electrotherapy, Interferential Current [TIAB] OR Acupunctures, Ear [TIAB] OR Ear Acupunctures [TIAB] OR Acupuncture*, Auricular [TIAB] OR Auricular Acupuncture* [TIAB] OR Ear Acupuncture [TIAB] OR Auriculotherap*[TIAB])

#3 ("Music Therapy”) [MH] OR Music Therapy [TIAB] OR Therapy, Music [TIAB]

#4 (Hypnosis [MH] OR Hypnosis [TIAB] OR Hypnotism [TIAB] OR Mesmerism [TIAB] OR Hypnotherapy [TIAB] OR Hypnotherapies [TIAB] OR Clinical Hypnosis [TIAB] OR Hypnosis, Clinical [TIAB] ORHypnoanalysis [TIAB] OR Self-Hypnosis [TIAB] OR Self Hypnosis [TIAB] OR Autohypnosis [TIAB])

#5 (Massage [MH] OR Massage [TIAB] OR Zone Therap* [TIAB] OR Therap*, Zone [TIAB] OR Massage Therap* [TIAB] OR Therap*, Massage [TIAB])

#6 (Mindfulness [MH] OR Mindfulness [TIAB] OR Meditation[TIAB])

#7 ("Cognitive Behavioral Therapy") [MH] OR Cognitive Behavioral Therapy [TIAB] OR Behavioral Therap*, Cognitive [TIAB] OR Cognitive Behavioral Therapies [TIAB] OR Therap*, Cognitive Behavioral [TIAB] OR Cognition Therap* [TIAB] OR Therapies, Cognition [TIAB] OR Therap*, Cognitive Behavior [TIAB] OR Behavior Therap*, Cognitive [TIAB] OR Cognitive Behavior Therap* [TIAB] OR Therapy, Cognition [TIAB] OR Cognitive Psychotherap* [TIAB] OR Psychotherap*, Cognitive [TIAB] OR Therap*, Cognitive [TIAB] OR Cognitive Therapies [TIAB] OR Cognitive Behaviour Therap* [TIAB] OR Behaviour Therap*, Cognitive [TIAB] OR Therap*, Cognitive Behaviour [TIAB] OR Cognitive Therapy [TIAB]

#8 ("Reality, Virtual")[MH] OR Reality, Virtual [TIAB]OR Virtual Reality, Educational [TIAB] OR Educational Virtual Realit* [TIAB] OR Reality, Educational Virtual [TIAB] OR Virtual Realit*, Educational [TIAB] OR Instructional Virtual Realit* [TIAB] OR Realit*, Instructional Virtual [TIAB] OR Virtual Realities, Instructional [TIAB]

#9 (Yoga [MH] OR Yoga[TIAB])

#10 (Qigong [MH] OR Qigong [TIAB] OR Ch'i Kung [TIAB] OR Qi Gong [TIAB] OR Taichi[TIAB] OR Tai Chi [TIAB] OR Taiji[TIAB] OR TAI JI [TIAB])

#11 ("Complementary Therapies" [MH] OR Complementary Therapies [TIAB] OR Therapies, Complementary [TIAB] OR Therapy, Complementary [TIAB] OR Alternative Medicine [TIAB] OR [TIAB] OR Medicine, Alternative [TIAB] OR Complementary Medicine [TIAB] OR Medicine, Complementary [TIAB] OR Alternative Therapies [TIAB] OR Therapies, Alternative [TIAB] OR Therapy, Alternative [TIAB])

#12 (Telephone [MH] OR Telephone* [TIAB] OR Switchboard Service* [TIAB] OR Service*, Switchboard [TIAB])

#13 Psychological therap* [TIAB] OR Spiritual intervention [TIAB]

#14 ("Analgesics, Opioid" [MH] OR Analgesics, Opioid [TIAB] OR Opioid Analgesic* [TIAB] OR Analgesic, Opioid [TIAB] OROpioid* [TIAB] OR Full Opioid Agonists [TIAB] OR Agonists, Full Opioid [TIAB] OR Opioid Agonists, Full [TIAB] OR Opioid Full Agonists [TIAB] OR Agonists, Opioid Full [TIAB] OR Full Agonists, Opioid [TIAB] OR Opioid Mixed Agonist-Antagonists [TIAB] OR Agonist-Antagonists, Opioid Mixed [TIAB] OR Mixed Agonist-Antagonists, Opioid [TIAB] OR Opioid Mixed Agonist Antagonists [TIAB] OR Partial Opioid Agonists [TIAB] OR Agonists, Partial Opioid [TIAB] OR Opioid Agonists, Partial [TIAB] OR Opioid Partial Agonists [TIAB] OR Agonists, Opioid Partial [TIAB] OR Partial Agonists, Opioid [TIAB])

#15 ("Medicine, Traditional")[MH] OR Medicine, Traditional [TIAB] OR Traditional Medicine [TIAB] OR Medicine, Folk [TIAB] OR Folk Medicine [TIAB] OR Medicine, Indigenous [TIAB] OR Indigenous Medicine [TIAB] OR Ethnomedicine [TIAB] OR Folk Remedies [TIAB] OR Folk Remedy [TIAB] OR Remedies, Folk [TIAB] OR Remedy, Folk [TIAB] OR Home Remedies [TIAB] OR Home Remedy [TIAB] OR Remedies, Home [TIAB] OR Remedy, Home [TIAB] OR Medicine, Primitive [TIAB] OR Primitive Medicine [TIAB]

#16 (#2 OR #3 OR #4 OR #5 OR #6 OR #7 OR #8 OR #9 OR #10 OR #11 OR #12 OR #13 OR #14 OR #15)

#17 (#1 AND #16)

#18 ("Clinical Trials as Topic" [MH] OR randomized controlled trial[Publication Type] OR controlled clinical trial[Publication Type] OR randomized[TIAB] OR placebo[TIAB] OR randomly[TIAB] OR trial[TIAB])

#19 (#17 AND #18)
