# Supplementary material for: Efficacy of pharmacological and non-pharmacological therapy on chronic cancer pain intensity of adults with cancer: A network meta-analysis protocol
Source: PLoS One. 2025 Jul 17;20(7):e0322651. doi: 10.1371/journal.pone.0322651 (PMC12270095; doi:10.1371/journal.pone.0322651)
Supplement: Attachment — Submitted filename: Response to Reviewers.docx [file pone.0322651.s009.docx]

Dear editor and reviewers:

On behalf of my co-authors, we appreciate editor and reviewers very much for their constructive comments and suggestions on our manuscript entitled “Efficacy of Pharmacological and Non-pharmacological Therapy on Chronic Cancer Pain Intensity of Adults with Cancer: A Network Meta-analysis Protocol”

We have studied the comments carefully and made corrections which we hope meet with approval. The corrections are in the manuscript and the responds to the reviewers’ comments are as follows**:**

**Replies to academic editor’s comments：**

**Academic editor #**

**General:**

**Comment 1: Ensure all actions are written in future tense.**

*Response:* *Thank you for your comment. Yes, we have changed our written tense in the new manuscript and carefully checked the future tense.*

**Comment 2: Thoroughly review and improve language throughout the manuscript.**

*Response: Yes, we improved our logic and language of the whole manuscript.*

**Abstract:**

**Comment 3: Rewrite the abstract to follow a clear and logical sequence, using PRISMA-NMA guidelines.**

*Response: We followed the PRISMA-NMA to rewrite the abstract, and here is the revised abstract.*

*Abstract*

*Background*

*Chronic cancer pain is very common symptom in cancer patients, but this issue has not been satisfactorily resolved by the conventional three-step analgesic therapy. There are multiple non-pharmacological interventions for managing chronic cancer pain, but we haven’t reached a consensus on which non pharmacological treatment is the best and these treatments are lack of high-quality evidence. In order to identify the most effective non-pharmaceutical therapy alternatives and investigate further possible medication interventions, this study will use network meta-analysis to assess the therapeutic effects of pharmacological and non-pharmacological treatments on chronic cancer pain patients and support clinical decision-making by prioritizing therapies according to the most valuable clinical outcomes for these patients.*

*Methods and analysis*

*We will carry out a systematic search of published randomized controlled trials (group, crossover, and parallel) in the PubMed, Web of Science, Cochrane Library, MEDLINE, Embase, and CINAHL databases, without language or date restrictions, in accordance with the PRISMA for Network Meta-Analyses (PRISMA-NMA) guidelines. Included studies must evaluate the effects of pharmacological and non-pharmacological treatments in patients with chronic cancer pain. Adult chronic cancer pain patients (≥ 18 years old) receiving pharmacological or non-pharmacological treatment will be our target participants. Our primary outcomes will be pain intensity, total effective rate of treatment, onset time, and quality of Life (QoL); Adverse reaction will be our secondary outcome. We'll utilize the mean difference (MD) for continuous variables, the odds ratio (OR) for binary variables, and the 95% confidence interval (CI) for interval estimates. The Cochrane Bias Risk Tool (RoB2.0) will be used to assess the bias risk of every RCT trial included in NMA. We will use Review Manager 5.3 software to conduct heterogeneity testing and meta-analysis. The network meta-analysis will be performed by ADDIS1.16.8 software. The Confidence in Network Meta-analysis (CINeMA) framework will be used to evaluate the level of confidence in the NMA results. Besides, we will use SUCRA for ranking the network meta-analysis results, and we will also apply normalized entropy to verify the accuracy of the SUCRA ranking outcomes.*

*Discussion*

*This network meta-analysis will compare the efficacy of pharmacological versus non-pharmacological treatments for pain intensity in chronic cancer pain patients. The final analysis results may be significantly heterogeneous, because the population with cancerous pain suffers from different types of cancers. Owing to the database's primary reliance on our listed databases for inclusion, potentially valuable research will be overlooked.*

*Registration*

*This study has been registered in the PROSPERO database (*[*CRD42024505214*](https://www.crd.york.ac.uk/prospero/display_record.php?ID=CRD42024496248)*).*

*Data statement*

*Deidentified research data will become publically available upon completion of the study and publication.*

*Funding*

*The author(s) received no specific funding for this work.*

**Comment 4: Include all study objectives, such as the total effective rate of treatment.**

*Response: We included all study objectives, primary outcomes include pain intensity, total effective rate of treatment, onset of analgesia, quality of life; secondary outcome is adverse reactions.*

**Comment 5: Briefly describe the statistical model and data analysis procedures.**

*Response: Thank you for your comment. We have added a brief description of the statistical model and data analysis procedures to provide clarity. Here is the revised manuscript.*

*“We will use ADDIS v1.16.8 software for meta-analysis and heterogeneity assessment. For dichotomous data, we will analyze using odds ratios (OR); for continuous variables, we will use mean differences (MD). A 95% confidence interval (CI) will be used to estimate the range of values. The Q test will be used to detect heterogeneity among studies, and the I² statistic will be used to assess the degree of heterogeneity. If I²≥50% and p≤0.10, a random-effects model will be used for analysis. Conversely, if I²<50% and p>0.10, a fixed-effects model will be used.*

*We will use the GeMTC package in R software to conduct Bayesian network meta-analysis (NMA) of the selected randomized controlled trials (RCTs). The node-splitting model (NM) will be used to check the consistency of the evidence network. If the observed differences are not statistically significant (P>0.05), a consistency model (CM) will be used for the NMA; if the differences are statistically significant (P<0.05), an inconsistency model (IM) will be used. We will run 50,000 simulation iterations and 10 burn-in iterations, and use the potential scale reduction factor (PSRF) to assess model convergence. When the PSRF value is close to or equal to 1, it indicates strong model convergence and reliable consistency model results.* *The surface under the cumulative ranking curve (SUCRA) will be calculated to determine the effectiveness of each intervention. SUCRA values range from 0 to 1, with a value of 1 indicating complete effectiveness and a value of 0 indicating no effectiveness. Interventions will be ranked based on their SUCRA values.*

*For dichotomous outcomes, we will use odds ratios (OR) to measure the relative effects between different interventions. For continuous outcomes, we will use standardized mean differences (SMD). We will use 95% confidence intervals (CI) to represent the estimated range of effect sizes. If the 95% CI does not include the null value (e.g., 1 for OR, 0 for SMD), the difference will be considered statistically significant. Additionally, we will use a p-value <0.05 as the criterion for statistical significance.*

*We will use the CINeMA framework to assess the credibility of the NMA results. CINeMA extends the GRADE (Grading of Recommendations, Assessment, Development, and Evaluations) approach by considering six domains to evaluate the certainty of evidence: within-study bias, reporting bias, indirectness, imprecision, heterogeneity, and incoherence. Evaluating the certainty of evidence through these methods will enhance the transparency, reproducibility, and reliability of the results. Two authors (S. W. and L. X.) will independently judge the quality of evidence (high, moderate, low, or very low), and any disagreements will be resolved by a third reviewer (W. Y.).”*

**Comment 6: Add protocol registration codes.**

*Response: This study has been registered in the PROSPERO database (*[*CRD42024505214*](https://www.crd.york.ac.uk/prospero/display_record.php?ID=CRD42024496248)*).*

**Introduction:**

**Comment 7. Define the "three-step therapy for pain relief."**

*Response: We added the definition of “three-step therapy for pain relief”. Here are the sentences in the revised manuscript.*

*“The three-step pain ladder, widely used in medical facilities, can alleviate pain in over 70% of cancer patients(6-8). The ladder consists of: (1) oral administration; (2) administering on time; (3) administering in steps; and (4) individualizing medication dosages. Based on the patient’s pain level and underlying cause, physicians choose appropriate analgesics. For first-grade (mild) pain, NSAIDs or acetaminophen with or without adjuvants are used. For second-grade (moderate) pain, weak opioids (hydrocodone, codeine, tramadol) are used with or without non-opioid analgesics and adjuvants. For third-grade (severe) pain, potent opioids (morphine, methadone, fentanyl, oxycodone, buprenorphine, tapentadol) are administered with or without non-opioid analgesics and adjuvants(8, 9). Additionally, the dosage of analgesics should be progressively increased from weak to strong(10).”*

**Comment 8: Revise the discussion of pharmacological dependence and adverse effects to ensure clarity and logical flow.**

*Response: After screening the literature and consulting with clinical doctors, we found that long-term use of opioid drugs strictly prescribed by doctors does not cause addiction problems in cancer patients, and the side effects of opioid drugs are more significant. Therefore, we will change the description to the side effects of long-term use of opioid drugs. Here is the revised sentences below.*

*“The adverse effects of opioid medications, such as opioid-induced neurotoxicity (15), constipation (16), nausea (17), and respiratory depression (18), present a significant challenge in managing chronic cancer pain (11). Opioids may also lower the immune system's efficacy, compromising anti-cancer therapies (14). They can suppress T lymphocytes and natural killer cells, impairing immune function and increasing infection rates (14). As cancer patients, particularly those with advanced cancer, require higher analgesic dosages and have lower health-related quality of life (13), non-pharmacological therapies have been recommended by the National Comprehensive Cancer Network (NCCN) clinical practice guidelines for adult cancer pain (19).”*

**Comment 9: Correct the term to "network meta-analysis."**

*Respo*nse: *Yes, we have corrected the term into the “network meta-analysis”.*

**Comment 10: Strengthen the argument to clearly support the research aims.**

*Response: Our research aim is to identify the most effective non-pharmaceutical therapy and investigate further possible medication interventions for cancer patients. Here is our revised argument:*

*“Chronic cancer pain is very common symptom in cancer patients, but this issue has not been satisfactorily resolved by the conventional three-step analgesic therapy. There are multiple non-pharmacological interventions for managing chronic cancer pain, but we haven’t reached a consensus on which non pharmacological treatment is the best and these treatments are lack of high-quality evidence.”*

**Methods:**

**Comment 11: Structure the content using the PRISMA-NMA extension.**

*Response: We rewrite the manuscript following the PRISMA-NMA extension guideline.*

**Comment 12: Include a summary of the eligibility criteria (PICOTS) within the manuscript.**

*Response: We have reset the eligibility criteria (PICOS). Here is the content in the revised manuscript.*

*“In this network meta-analysis, we will include participants diagnosed with chronic cancer pain, according to the criteria defined by the International Association for the Study of Pain (IASP)(33). While it is generally accepted that chronic pain is defined as pain lasting longer than three months, the precise concept of time is not stressed in cancer pain because cancer patients may experience rapid disease progression and shortened survival times. Therefore, we will define chronic cancer pain is a kind of chronic pain caused by the primary cancer itself or metastases (chronic cancer pain) or its treatment (chronic postcancer treatment pain) (33).*

*We will include randomized controlled trials (RCTs) that feature a control group for efficacy comparison. The control group can receive a placebo, conventional treatment, or any intervention different from the primary treatment method. If a study lacks a control group, it must demonstrate that the target intervention has higher efficacy compared to other interventions applied in the sample.*

*The studies must employ a randomized controlled trial design to assess the effectiveness of both pharmacological and non-pharmacological treatments for chronic cancer pain patients. Non-pharmacological interventions must not impose any pharmacological effects on participants.*

*Participants must be adult males or females, at least 18 years old, experiencing pain caused by cancer. Both race and gender will be considered independent variables in the research”.*

| **Table 1. Summary of PICOS eligibility criteria** | |
| --- | --- |
|  | **Inclusion criteria** |
| **Population** | Adults with chronic cancer pain diagnosed with IASP **criteria** |
| **Intervention** | Non-pharmacological interventions |
| **Comparison** | pharmacological interventions, placebo, sham, no intervention |
| **Outcome** | Pain intensity, total effective rate of treatment, quality of life, adverse reaction |
| **Study design** | Randomized controlled trials (RCTs) |

**Comment 13: Define the diagnostic criteria for the sample.**

*Response: we will define chronic cancer pain is a kind of chronic pain caused by the primary cancer itself or metastases (chronic cancer pain) or its treatment (chronic postcancer treatment pain).*

**Comment 14: Describe the types of randomized clinical trials to be included.**

*Response: We will include parallel groups, cross-over, and cluster randomized controlled trials. In crossover designs, only data from the initial trial period will be retrieved to exclude any potential carryover effects. All included studies must have a minimum of two different intervention measures.*

**Comment 15: Clarify that subjects with pain not related to cancer will be excluded.**

*Response: A reviewer suggested PICOS has already made it clear of exclusion criteria, and we think it’s not necessary to point that subjects with pain not related to cancer will be excluded.*

**Comment 16: Clearly define the pre-defined intervention sets based on published guidelines and systematic reviews.**

*Response: We created the pre-defined intervention node file (additional file S2) based on published guidelines and systematic reviews.*

**Comment 17:** **Create separate control groups for "placebo+sham," "no intervention+waiting list," and "standard drug treatment" to ensure transitivity.**

*Response: Thank you for your helpful advice, here is the revised sentences in the revised manuscript.*

*“Each intervention mentioned above controls for completely different confounding factors, so we will create three different control groups that include "placebo & sham," "no intervention & waiting list," and "standard drug treatment." To ensure that various interventions do not violate the assumption of transitivity for the network meta-analysis (NMA), we will define the "standard drug treatment" as following the three-step pain ladder characterized by nonsteroidal anti-inflammatory drugs, weak opioids, and strong opioids. In this network meta-analysis, comparisons of interventions will be made with each other.”*

**Comment 18: Define criteria for the "standard drug treatment" group to maintain consistency.**

*Response: We added the words to define criteria for the "standard drug treatment" group to maintain consistency.*

*“We will define the definition of "standard drug treatment". It will be defined as “the three-step pain ladder” which characterized by nonsteroidal anti-inflammatory drugs, week opioids, and strong opioids. To make it clear for this network meta-analysis, comparisons of interventions will be compared with each other. All control groups in the studies we will include will be assigned to these three groups.”*

**Comment 19: Clearly state primary and secondary outcomes in separate paragraphs.**

*Response: We separated the primary and secondary outcomes in different paragraphs：*

*“Outcomes*

*The results will be categorized into primary and secondary outcomes. We will include studies that assessed at least one of the following indicators:*

*Primary outcomes*

*Pain intensity*

*We will prioritize obtaining data from a single-dimensional pain scale. We will prioritize extracting numerical rating scale (NRS) data. If the studies included in the analysis do not utilize NRS, we will include the data in the following order: visual analog scale (VAS), verbal rating scale (VRS), verbal descriptor scale (VDS), Wong Baker faces pain scale revision (FPS-R), and Mankoski pain scale (MPS). The examination of multidimensional pain tools will only be considered if there is no alternative tool available for a single dimensional pain scale. We are going to use the multi-dimensional assessment tool in following orders if there is no single-dimensional pain scale being used in the included trials: brief pain inventory (BPI), short-form McGill pain questionnaire (SF/MPQ), and global pain scale (GPS). We will prioritize extracting the average pain, mildest pain, most severe pain, and pain data during rest and activity over the past 24 hours. The pain intensity assessment tool will be described in the trial's characteristics.*

*Total effective rate of treatment*

*If the study does not clearly disclose the total effective rate of treatment data, we will calculate them by hand. To calculate the total effective rate of treatment, we will divide the total number of effective treatments in the study by the total number of interventions. A effective treatment is defined as a reduction of at least 40% in patient pain intensity or frequency before and after treatment. The total effective rate of the treatment assessment tool will be described in the trial's characteristics.*

*Onset of analgesia*

*The onset of analgesia will be identified by assessing the time it takes for patients to experience substantial pain alleviation or complete elimination of pain following analgesic treatment.*

*Quality of life*

*We will prioritize collecting data from the European Organization for Research and Treatment of Cancer Quality of Life Questionnaire 30-Item Core Instrument (EORTC QLQ-C30). If the EORTC QLQ-C30 cannot be obtained from the studies included, we will collect data from the Functional Assessment of Cancer Therapy General Scale (FACT-G). We will also include other quality of life assessment tools, and quality of life assessment tool will be listed in the trial's characteristics as well.*

*Secondary outcome*

*Adverse reactions*

*As secondary outcome indicators, we will include treatment reactions such as nausea, vomiting, constipation, respiratory depression, fluctuations in blood pressure, and death. Since objective techniques cannot accurately measure subjective symptoms, we will exclude subjective symptom measurement instruments from the report. For measurable adverse reactions such as blood pressure, we will report them in the adverse reactions section.”*

**Comment 20: Develop a detailed description of data extraction variables for methods, participants, interventions, and outcomes.**

*Response:*

*Thank you for your comment. We have developed a detailed description of data extraction variables for methods, participants, interventions, and outcomes*

**Comment 21: Address the assumption of transitivity and coherence.**

*Response:* *Thank you for your comment. We have addressed the assumption of transitivity and coherence to ensure the validity and interpretability of the effect estimates.*

**Comment 22: Include SUCRA analysis for evaluating relative treatment effectiveness.**

*Response: We added the SUCRA analysis in the “Strategy for data synthesis and analysis” section.*

*Here are the sentences in the manuscript.*

*“The surface under the cumulative ranking curve (SUCRA) will be calculated to determine the effectiveness of each intervention. SUCRA values range from 0 to 1, with a value of 1 indicating complete effectiveness and a value of 0 indicating no effectiveness. Interventions will be ranked based on their SUCRA values.”*

**Discussion:**

**Comment 23: Reiterate that the protocol was written following the PRISMA-P and PRISMA-NMA.**

*Response: Thank you for your kind suggestion, we have corrected it in “Materials and methods” section. Here is the revised manuscript below.*

*“We have completed the PRISMA-NMA(S1 File) and PRISMA-P(S2 File).”*

**Comment 24: Conclude by emphasizing the lack of studies directly comparing interventions for cancer pain.**

*Response: In the last paragraph, we emphasized the ack of studies directly comparing interventions for chronic cancer pain.*

*“Currently, there is a lack of research explicitly comparing various chronic cancer pain interventions”*

**Replies to reviewer 1’s comments：**

**Reviewer 1#**

**ABSTRACT**

**Comment 1:**

**（1）-The writing is confusing and lacks an expected logical sequence. I suggest incorporating the use of PRISMA-NMA as a criterion for reporting the information in this manuscript.**

*Response: We followed the PRISMA-NMA to write our protocol.*

**（2）- I suggest including the other objectives of the study in the abstract, such as “total effective rate of treatment”.**

*Response: Thank you for your very helpful advice, we added all our outcomes, registration codes in the abstract. Here is the revised manuscript below.*

*“Our primary outcomes will be pain intensity, total effective rate of treatment, onset time, and quality of Life (QoL); Adverse reaction will be our secondary outcome.”*

**（3）- Statistical model must be briefly described in the methods, in addition to which procedures will be followed during data extraction and analysis.**

*Response:* *Thank you for your helpful advice. We have made our corrections in the manuscript below.*

“*This network meta-analysis protocol is registered with PROSPERO (CRD42024505214) and will follow the PRISMA-NMA extension statement. We will include randomized controlled trials (RCTs) involving adults (≥18 years old) diagnosed with chronic cancer pain according to the criteria defined by the International Association for the Study of Pain (IASP). Eligible studies must compare the efficacy of pharmacological and non-pharmacological interventions for chronic cancer pain. Non-pharmacological interventions should not impose pharmacological effects on participants. The primary outcomes will include pain intensity, overall treatment efficacy, quality of life, and adverse reactions.*

*We will conduct a comprehensive literature search in databases such as PubMed, Web of Science, Cochrane Library, MEDLINE, Embase, and CINAHL, without language or date restrictions. Grey literature will also be investigated. Data extraction will be performed independently by two reviewers, with discrepancies resolved by a third reviewer. We will assess the quality of included studies using the Cochrane risk-of-bias tool. A network meta-analysis will be conducted to compare multiple interventions simultaneously, considering both direct and indirect evidence. Statistical analysis will be performed using R and ADDIS software.”*

**（4）- I believe it is important to add the protocol registration codes.**

*Response: This study has been registered in the PROSPERO database (*[*CRD42024505214*](https://www.crd.york.ac.uk/prospero/display_record.php?ID=CRD42024496248)*).*

**INTRODUCTION**

**Comment 2:**

**- “Today, the principles of three-step therapy for pain relief are often used in clinical practice”, what are they?**

*Response: We defined the three-step therapy as follows*

*“The three-step pain ladder, widely used in medical facilities, can alleviate pain in over 70% of cancer patients (6-8). The ladder consists of: (1) oral administration; (2) administering on time; (3) administering in steps; and (4) individualizing medication dosages. Based on the patient’s pain level and underlying cause, physicians choose appropriate analgesics. For first-grade (mild) pain, NSAIDs or acetaminophen with or without adjuvants are used. For second-grade (moderate) pain, weak opioids (hydrocodone, codeine, tramadol) are used with or without non-opioid analgesics and adjuvants. For third-grade (severe) pain, potent opioids (morphine, methadone, fentanyl, oxycodone, buprenorphine, tapentadol) are administered with or without non-opioid analgesics and adjuvants (8, 9). Additionally, the dosage of analgesics should be progressively increased from weak to strong (10).”*

**Comment 3:**

**- “although treating cancer pain still presents difficult obstacles posed by opioid analgesic addiction and pharmacological adverse effects. Patients of late-stage cancer are less likely to become addicted to painkillers because they are less likely to acquire pharmacological resistance, requiring higher dosages to manage pain in later stages of the disease”**

*First, the problem of “pharmacological dependence and adverse effects” is addressed. Subsequently, the argument is broken with the information that patients in advanced stages are LESS likely to acquire resistance to drugs, which is why they need to be exposed to high doses.*

*Wouldn't they be MORE likely? I suggest reviewing your writing, with the aim of connecting the information and creating a logical sense in the story you want to develop. Reading is not fluid!*

*Response: Thank you for your comments. We have discussed this question seriously. After talking and inquiring clinical doctors, we realized that the long-term use of opioid will not be easily get addicted under doctor’s careful diagnoses and treatments. So we changed this part discussion. We decided to have more discussion about the adverse reaction of long-term use of opioid medicine. Here is our new writing in the revised manuscript. We hope this can meet your comment.*

*“Alleviating chronic cancer pain has become a crucial clinical issue. The three-step pain ladder, widely used in medical facilities, can alleviate pain in over 70% of cancer patients (6-8). The ladder consists of: (1) oral administration; (2) administering on time; (3) administering in steps; and (4) individualizing medication dosages. Based on the patient’s pain level and underlying cause, physicians choose appropriate analgesics. For first-grade (mild) pain, NSAIDs or acetaminophen with or without adjuvants are used. For second-grade (moderate) pain, weak opioids (hydrocodone, codeine, tramadol) are used with or without non-opioid analgesics and adjuvants. For third-grade (severe) pain, potent opioids (morphine, methadone, fentanyl, oxycodone, buprenorphine, tapentadol) are administered with or without non-opioid analgesics and adjuvants (8, 9). Additionally, the dosage of analgesics should be progressively increased from weak to strong (10).*

*However, the adverse effects of opioid medications, such as opioid-induced neurotoxicity (15), constipation (16), nausea (17), and respiratory depression (18), present a significant challenge in managing chronic cancer pain (11). Opioids may also lower the immune system's efficacy, compromising anti-cancer therapies (14). They can suppress T lymphocytes and natural killer cells, impairing immune function and increasing infection rates (14). As cancer patients, particularly those with advanced cancer, require higher analgesic dosages and have lower health-related quality of life (13), non-pharmacological therapies have been recommended by the National Comprehensive Cancer Network (NCCN) clinical practice guidelines for adult chronic cancer pain (19).*

*Despite guidelines recommending opioid medications (2), inadequate treatment is still widespread, with 32% to 82.3% of patients not receiving adequate pain management (20). This is due to factors such as difficult access to medication for at-home care, overly cautious prescribing by clinicians (21), and strict regulations on analgesic use in some countries. The focus of chronic cancer pain treatment guidelines primarily on pharmacological therapy often neglects the specificity of elderly patients with advanced cancer, who may experience adverse effects from comorbidities and multiple medications.”*

**Comment 4:**

**- “net meta-analysis” Correct term in the 28th line of page 2.**

*Response: Thank you for your comment. We corrected the “net meta-analysis” into “network meta-analysis”.*

**Comment 5:**

**Arguments are not coherent, making it difficult for the reader to perceive the importance of the study.**

*Response:*

*Thank you for your feedback. We have revised the manuscript to reorganize the arguments, ensuring better coherence and making it easier for readers to grasp the importance of the study. We hope these improvements meet your expectations.*

**METHOD**

**Comment 6:**

**- If it was not used, I suggest using the PRISMA-NMA extension to structure the contents of this systematic review and network meta-analysis.**

*Response: Thank you for your advice. We rewrote the manuscript based on PRISMA-NMA extension.*

**Comment 7: Eligibility criteria**

**- I suggest placing the summary of the eligibility criteria (PICOTS) within the manuscript, to facilitate reader understanding.**

*Response: We redefined the population and the PICOS table. Here are the criteria in the manuscript.*

*“In this network meta-analysis, we will include participants diagnosed with chronic cancer pain, according to the criteria defined by the International Association for the Study of Pain (IASP)(33). While it is generally accepted that chronic pain is defined as pain lasting longer than three months, the precise concept of time is not stressed in chronic cancer pain because cancer patients may experience rapid disease progression and shortened survival times. Therefore, we will define chronic cancer pain is a kind of chronic pain caused by the primary cancer itself or metastases (chronic cancer pain) or its treatment (chronic postcancer treatment pain) (33).*

*We will include randomized controlled trials (RCTs) that feature a control group for efficacy comparison. The control group can receive a placebo, conventional treatment, or any intervention different from the primary treatment method. If a study lacks a control group, it must demonstrate that the target intervention has higher efficacy compared to other interventions applied in the sample.*

*The studies must employ a randomized controlled trial design to assess the effectiveness of both pharmacological and non-pharmacological treatments for chronic cancer pain patients. Non-pharmacological interventions must not impose any pharmacological effects on participants.*

*Participants must be adult males or females, at least 18 years old, experiencing pain caused by cancer. Both race and gender will be considered independent variables in the research.”*

| ***Table 1. Summary of PICOS eligibility criteria*** | |
| --- | --- |
|  | *Inclusion criteria* |
| *Population* | *Adults with chronic cancer pain diagnosed with IASP criteria* |
| *Intervention* | *Non-pharmacological interventions* |
| *Comparison* | *pharmacological interventions, placebo, sham, no intervention* |
| *Outcome* | *Pain intensity, total effective rate of treatment, quality of life, adverse reaction* |
| *Study design* | *Randomized controlled trials (RCTs)* |

**Comment 8: Population**

**- I believe it is important to define which diagnostic criteria will be used to define the sample.**

*Response: We have written the criteria of chronic cancer pain. Here is the revised population definition.*

*“In this network meta-analysis, we will include participants diagnosed with chronic cancer pain, according to the criteria defined by the International Association for the Study of Pain (IASP)(33). While it is generally accepted that chronic pain is defined as pain lasting longer than three months, the precise concept of time is not stressed in cancer pain because cancer patients may experience rapid disease progression and shortened survival times. Therefore, we will define chronic cancer pain is a kind of chronic pain caused by the primary cancer itself or metastases (chronic cancer pain) or its treatment (chronic postcancer treatment pain) (33).”*

**Comment 9:**

**- What types of clinical trials will be included? I suggest describing the possible types of randomized clinical trials, for example parallel group, cross-over or cluster RCT.**

*Response: We rewrote the clinical trials inclusion criteria. Here is the new criteria.*

*“We will include randomized controlled trials (RCTs) that feature a control group for efficacy comparison. The control group can receive a placebo, conventional treatment, or any intervention different from the primary treatment method. If a study lacks a control group, it must demonstrate that the target intervention has higher efficacy compared to other interventions applied in the sample.*

*The studies must employ a randomized controlled trial design to assess the effectiveness of both pharmacological and non-pharmacological treatments for chronic cancer pain patients. Non-pharmacological interventions must not impose any pharmacological effects on participants.*

*Participants must be adult males or females, at least 18 years old, experiencing pain caused by cancer. Both race and gender will be considered independent variables in the research.”*

**Comment 10:**

**- “The subjects of the research include those with pain that is not related to cancer”. I think the correct would be “DO NOT include”.**

*Response: yes, we corrected this sentence.*

**Comment 11:**

**Interventions**

**- How were the intervention sets to be included pre-defined? I recommend using previously published clinical guidelines and systematic reviews as a basis. I suggest searching the literature for recently published articles, with the aim of creating reliable and relevant nodes for the condition of interest.**

*Response: Yes, we followed your helpful advice and created an intervention measure file(S3) based on the previously published clinical guidelines, RCTs and systematic reviews.*

**Comment 12:**

**Comparator**

**- I believe it is not appropriate to incorporate the interventions into a single node: standard drug treatment, placebo and no intervention. Each intervention above controls for completely different confounding factors. Such a decision would violate the principle of transitivity, the primary criterion for conducting network meta-analysis. I suggest creating three different control groups, "placebo+sham", "no intervention+waiting list" and "standard drug treatment".**

*Response: We have followed your suggestion, and made the corrections below. We removed the “Comparator” and put this part in the “Intervention”.*

*“Each intervention mentioned above controls for completely different confounding factors, so we will create three different control groups that include "placebo & sham," "no intervention & waiting list," and "standard drug treatment." To ensure that various interventions do not violate the assumption of transitivity for the network meta-analysis (NMA), we will define the "standard drug treatment" as following the three-step pain ladder characterized by nonsteroidal anti-inflammatory drugs, weak opioids, and strong opioids. In this network meta-analysis, comparisons of interventions will be made with each other. All control groups in the studies we will include will be assigned to these three groups.”*

**Comment 13:**

**- Regarding the "standard drug treatment" group, it is necessary to be aware of the lack of consistency in its definition in clinical trials. Including different intervention modalities in the same node can potentially violate the assumption of transitivity for the NMA. I suggest creating criteria for the use of this group.**

*Response: We followed your suggestion, and make the correction below:*

*“We will define the definition of "standard drug treatment". It will be defined as “the three-step pain ladder” which characterized by nonsteroidal anti-inflammatory drugs, week opioids, and strong opioids. To make it clear for this network meta-analysis, comparisons of interventions will be compared with each other. All control groups in the studies we will include will be assigned to these three groups.”*

**Comment 14:**

**Outcomes**

**- Please clarify what the primary and secondary outcomes will be. It is unclear, will quality of life be a secondary outcome? I suggest placing primary outcomes and secondary outcomes in different paragraphs.**

*Response: Yes we have separated the and secondary outcomes in different paragraphs. Here are the corrections below:*

*Outcomes*

*The results will be categorized into primary and secondary outcomes. We will include studies that assessed at least one of the following indicators:*

*Primary outcomes*

*Pain intensity*

*We will prioritize obtaining data from a single-dimensional pain scale. We will prioritize extracting numerical rating scale (NRS) data. If the studies included in the analysis do not utilize NRS, we will include the data in the following order: visual analog scale (VAS), verbal rating scale (VRS), verbal descriptor scale (VDS), Wong Baker faces pain scale revision (FPS-R), and Mankoski pain scale (MPS). The examination of multidimensional pain tools will only be considered if there is no alternative tool available for a single dimensional pain scale. We are going to use the multi-dimensional assessment tool in following orders if there is no single-dimensional pain scale being used in the included trials: brief pain inventory (BPI), short-form McGill pain questionnaire (SF/MPQ), and global pain scale (GPS). We will prioritize extracting the average pain, mildest pain, most severe pain, and pain data during rest and activity over the past 24 hours. The pain intensity assessment tool will be described in the trial's characteristics.*

*Total effective rate of treatment*

*If the study does not clearly disclose the total effective rate of treatment data, we will calculate them by hand. To calculate the total effective rate of treatment, we will divide the total number of effective treatments in the study by the total number of interventions. A effective treatment is defined as a reduction of at least 40% in patient pain intensity or frequency before and after treatment. The total effective rate of the treatment assessment tool will be described in the trial's characteristics.*

*Onset of analgesia*

*The onset of analgesia will be identified by assessing the time it takes for patients to experience substantial pain alleviation or complete elimination of pain following analgesic treatment.*

*Quality of life*

*We will prioritize collecting data from the European Organization for Research and Treatment of Cancer Quality of Life Questionnaire 30-Item Core Instrument (EORTC QLQ-C30). If the EORTC QLQ-C30 cannot be obtained from the studies included, we will collect data from the Functional Assessment of Cancer Therapy General Scale (FACT-G). We will also include other quality of life assessment tools, and quality of life assessment tool will be listed in the trial's characteristics as well.*

*Secondary outcome*

*Adverse reactions*

*As secondary outcome indicators, we will include treatment reactions such as nausea, vomiting, constipation, respiratory depression, fluctuations in blood pressure, and death. Since objective techniques cannot accurately measure subjective symptoms, we will exclude subjective symptom measurement instruments from the report. For measurable adverse reactions such as blood pressure, we will report them in the adverse reactions section.*

**Comment 15: Study design**

**-“The list will only contain items that have been published in English”. I suggest not restricting your search by language. Controlling this bias will give more credibility to potentially identified estimates.**

*Response: yes, we adopted your suggestion, and there is no restriction on literature language and date. Here is our correction below:*

***“Eligibility Criteria***

*In this network meta-analysis, we will include participants diagnosed with chronic cancer pain, according to the criteria defined by the International Association for the Study of Pain (IASP)(33). While it is generally accepted that chronic pain is defined as pain lasting longer than three months, the precise concept of time is not stressed in cancer pain because cancer patients may experience rapid disease progression and shortened survival times. Therefore, we will define chronic cancer pain is a kind of chronic pain caused by the primary cancer itself or metastases (chronic cancer pain) or its treatment (chronic postcancer treatment pain) (33).*

*We will include randomized controlled trials (RCTs) that feature a control group for efficacy comparison. The control group can receive a placebo, conventional treatment, or any intervention different from the primary treatment method. If a study lacks a control group, it must demonstrate that the target intervention has higher efficacy compared to other interventions applied in the sample.*

*The studies must employ a randomized controlled trial design to assess the effectiveness of both pharmacological and non-pharmacological treatments for cancer pain patients. Non-pharmacological interventions must not impose any pharmacological effects on participants.*

*Participants must be adult males or females, at least 18 years old, experiencing pain caused by cancer. Both race and gender will be considered independent variables in the research.”*

| ***Table 1. Summary of PICOS eligibility criteria*** | |
| --- | --- |
|  | *Inclusion criteria* |
| *Population* | *Adults with chronic cancer pain diagnosed with IASP criteria* |
| *Intervention* | *Non-pharmacological interventions* |
| *Comparison* | *pharmacological interventions, placebo, sham, no intervention* |
| *Outcome* | *Pain intensity, total effective rate of treatment, quality of life, adverse reaction* |
| *Study design* | *Randomized controlled trials (RCTs)* |

**Comment 16:**

**Data extraction**

**- I suggest better developing the variables extracted from those eligible, separating specific information from the topics: methods, participants, interventions and outcomes. For example: Methods: study design, study setting, and year of publication; Participants: sample size, mean age, sex, disease duration, comorbidities, diagnostic criteria...**

**Strategy for data synthesis and analysis**

*Response:*

*Thank you for your valuable suggestions. We have refined the data extraction process by categorizing the variables into specific topics such as methods, participants, interventions, and outcomes. For instance, under methods, we now include study design, study setting, and year of publication; for participants, we detail sample size, mean age, sex, disease duration, comorbidities, and diagnostic criteria. These changes aim to enhance clarity and organization in our data synthesis and analysis strategy.*

**Comment 17:**

**- It is not clear whether the assumption of transitivity and coherence will be realized. If not programmed, I suggest adding it as a measure to ensure the validity and interpretability of the effect estimates found.**

*Response:*

*Thank you for your insightful comment. We have addressed the assumption of transitivity and coherence in our methodology to ensure the validity and interpretability of the effect estimates. These measures are integral parts of our analysis protocol. Here is our correction in the revised manuscript.*

*“To ensure that various interventions do not violate the assumption of transitivity for the network meta-analysis (NMA), we will define the "standard drug treatment" as following the three-step pain ladder characterized by nonsteroidal anti-inflammatory drugs, weak opioids, and strong opioids. In this network meta-analysis, comparisons of interventions will be made with each other. All control groups in the studies we will include will be assigned to these three groups.*

*We will use ADDIS v1.16.8 software for meta-analysis and heterogeneity assessment. For dichotomous data, we will analyze using odds ratios (OR); for continuous variables, we will use mean differences (MD). A 95% confidence interval (CI) will be used to estimate the range of values. The Q test will be used to detect heterogeneity among studies, and the I² statistic will be used to assess the degree of heterogeneity. If I²≥50% and p≤0.10, a random-effects model will be used for analysis. Conversely, if I²<50% and p>0.10, a fixed-effects model will be used.*

*We will use the GeMTC package in R software to conduct Bayesian network meta-analysis (NMA) of the selected randomized controlled trials (RCTs). The node-splitting model (NM) will be used to check the consistency of the evidence network. If the observed differences are not statistically significant (P>0.05), a consistency model (CM) will be used for the NMA; if the differences are statistically significant (P<0.05), an inconsistency model (IM) will be used. We will run 50,000 simulation iterations and 10 burn-in iterations, and use the potential scale reduction factor (PSRF) to assess model convergence. When the PSRF value is close to or equal to 1, it indicates strong model convergence and reliable consistency model results. The surface under the cumulative ranking curve (SUCRA) will be calculated to determine the effectiveness of each intervention. SUCRA values range from 0 to 1, with a value of 1 indicating complete effectiveness and a value of 0 indicating no effectiveness. Interventions will be ranked based on their SUCRA values.*

*For dichotomous outcomes, we will use odds ratios (OR) to measure the relative effects between different interventions. For continuous outcomes, we will use standardized mean differences (SMD). We will use 95% confidence intervals (CI) to represent the estimated range of effect sizes. If the 95% CI does not include the null value (e.g., 1 for OR, 0 for SMD), the difference will be considered statistically significant. Additionally, we will use a p-value <0.05 as the criterion for statistical significance.*

*We will use the CINeMA framework to assess the credibility of the NMA results. CINeMA extends the GRADE (Grading of Recommendations, Assessment, Development, and Evaluations) approach by considering six domains to evaluate the certainty of evidence: within-study bias, reporting bias, indirectness, imprecision, heterogeneity, and incoherence. Evaluating the certainty of evidence through these methods will enhance the transparency, reproducibility, and reliability of the results. Two authors (S. W. and L. X.) will independently judge the quality of evidence (high, moderate, low, or very low), and any disagreements will be resolved by a third reviewer (W. Y.).”*

**Comment 18:**

**- I suggest the use of SUCRA (Surface Under the Cumulative Ranking curve), with the aim of evaluating and classifying the relative effectiveness of different treatments in relation to the outcomes of interest.**

*Response: We have followed your advice and made our correction below:*

*“The surface under the cumulative ranking curve (SUCRA) will be calculated to determine the effectiveness of each intervention. SUCRA values range from 0 to 1, with a value of 1 indicating complete effectiveness and a value of 0 indicating no effectiveness. Interventions will be ranked based on their SUCRA values.”*

**Replies to reviewer 2’s comments：**

**Reviewer #2:**

**ABSTRACT**

**Comment 1: Remove the sentence "offering substantial proof so that decisions can be made with understanding" from the Background. It may be in the Discussion section, not in the Background.**

*Response: Yes, we agreed with your advice, and removed this sentence.*

**Comment 2: "Method" should be "Methods".**

*Response: We corrected “Method” into “Methods”*

**Comment 3: "in comparison to standard care, no intervention, or a placebo". * It's confusing to me. If it is a network meta-analysis, both studies with pharmacological and non-pharmacological treatments will be included, whether in comparison with another active or placebo. Is not true? I suggest that the sentence be rewritten to something like: "To be included in the review, studies must be randomized controlled trials evaluating the effects of pharmacological and non-pharmacological treatments in patients with cancer pain".**

*Response: Yes, we followed your advice, and rewrote this part in “Study design”. Here is our correction.*

*“We will include randomized controlled trials (RCTs) that feature a control group for efficacy comparison. The control group can receive a placebo, conventional treatment, or any intervention different from the primary treatment method. If a study lacks a control group, it must demonstrate that the target intervention has higher efficacy compared to other interventions applied in the sample.*

*The studies must employ a randomized controlled trial design to assess the effectiveness of both pharmacological and non-pharmacological treatments for chronic cancer pain patients. Non-pharmacological interventions must not impose any pharmacological effects on participants.*

*Participants must be adult males or females, at least 18 years old, experiencing pain caused by cancer. Both race and gender will be considered independent variables in the research.”*

**Comment 4: I suggest removing the phrase "We will follow the Preferred Reporting Items for Systematic Review and Meta-Analysis Protocols (PRISMA-P) 2015 checklist".**

*Response: As another reviewer suggests, we put PRISMA-P in the discussion. Here is our revised manuscript.*

*“we will adhere to the PRISMA-P and PRISMA-NMA guidelines in our study composition.”.*

**Comment 5. Add how the results will be presented: MD with credible intervals using a league table? Will treatments also be classified using the surface under the cumulative ranking curve (SUCRA) method? Make it clear to the reader.**

*Response:*

*Thank you for your comment. We have clarified that the results will be presented as standardized mean differences (SMD) with credible intervals, using a league table format. Additionally, treatments will be classified using the surface under the cumulative ranking curve (SUCRA) method to provide a clear and comprehensive interpretation.*

**Comment 6: The "Discussion" is poor. Improve, making clear its importance, given the scarcity of studies in this area, etc. Furthermore, the phrase "This network meta-analysis compares the efficacy of pharmacological versus non-pharmacological treatments for pain intensity in patients with cancer pain" should be in the future.**

*Response:* *Yes, the original manuscript has a poor discussion in this area, so we added more description about the current medicine therapy and its setbacks. Here are the new revised sentences below.*

*“Controlling and treating chronic cancer pain, particularly in late-stage patients, remains a significant health challenge. The standard three-step analgesic ladder often struggles to effectively address this issue. Our objective is to evaluate published RCT studies on chronic cancer pain interventions, and we will adhere to the PRISMA-P and PRISMA-NMA guidelines in our study composition. The CINeMA framework will be utilized to assess the credibility of our analysis, considering factors such as internal bias, indirectness, and uncertainty(45). We will use the ROB 2.0 tool to evaluate various aspects of internal bias in the studies, including intervention bias, missing outcome data, and randomization processes(46), and incorporate non-pharmacological interventions into our NMA.*

*Initial mild chronic cancer pain is typically treated with NSAIDs. As the disease progresses, high doses of potent opioid medications may be required. Prolonged use of these drugs can lead to severe adverse effects. Therefore, additional effective intervention strategies are needed beyond pharmacological treatments. To date, only two NMA studies(47, 48) have compared non-pharmacological interventions in chronic cancer pain management, and only one NMA studies(49) have compared pharmacological interventions. Acupuncture-point stimulation has the potential to alleviate moderate to severe cancer pain as well as minimize the negative effects of opioids, including nausea, vomiting, and constipation(47). The three-step pain ladder in conjunction with manual acupuncture is the most effective in terms of clinical efficacy and functional activity status improvement for patients. When considering the length of analgesic and analgesic duration, combined acupoint moxibustion produces the best results(48).After analyzing 81 randomized controlled trials, there are considerable variations in efficacy between existing regimens for chronic cancer pain. They found that certain nonopioid analgesics and nonsteroidal anti-inflammatory drugs can serve as effectively as opioids in managing chronic cancer pain. And in terms of pain intensity, they discovered that no pharmaceutical class significantly improved pain intensity compared to placebo, but the nonopioid analgesic ziconotide, the opioid dezocine, and the NSAID diclofenac were the most effective individual treatments(49). However, these studies did not encompass all available interventions, limiting their effect size and level of evidence. Our research aims to identify measures that can alleviate severe pain, surpassing the traditional three-step analgesic ladder, to improve patients' quality of life and pain control.*

*Our NMA has several advantages. We will conduct a comprehensive search of existing pharmacological and non-pharmacological interventions for chronic cancer pain and rank these treatments using SUCRA. Additionally, we will employ normalized entropy to verify the SUCRA ranking results, enhancing the reliability of our findings.*

*Currently, there is a lack of research explicitly comparing various chronic cancer pain interventions. We anticipate that our NMA will provide valuable treatment recommendations to healthcare professionals, researchers, and patients, assisting clinicians in making more informed decisions.”*

*We realized that the phrase "This network meta-analysis compares the efficacy of pharmacological versus non-pharmacological treatments for pain intensity in patients with cancer pain" is wrong, and we corrected it.*

**METHODS**

**Comment 1: The section must be "Methods" and not "Method".**

*Response:* *Yes, we corrected it.*

**Comment 2: In the "Population" section, remove "Inclusion criteria". Keep just "Population". The general section is already called "Eligibility criteria".**

*Response:* Thank you for your good advice, we removed the “Inclusion criteria”.

**Comment 3: I suggest "in the PROSPERO" instead of "with PROSPERO".**

*Response: Thank you for your helpful suggestion, we corrected it in the manuscript.*

**Comment 4: "The World Health Organization reports that individuals with cancerous pain who are 18 years of age or older will be included in the study. Chronic cancer pain is defined by the International Association for Pain Research (IASP) as persistent pain caused by primary cancer lesions, metastatic lesions, or cancer-related treatments".**

**- I didn't understand the meaning of that sentence. In the "Population" section, describe only the eligible criteria for the population of interest. For example:**

**Population: Adults (18 years or older) diagnosed with cancer... and so on. Furthermore, consider informing whether the inclusion criteria are independent of gender and race. The reader cannot be left in doubt.**

*Response: Thank you for your insightful advice. As we have more deep literature research, we find that there is not accurate definition of cancer pain, but chronic cancer pain. And we decided to use chronic cancer pain to describe our target population, because it’s more accurate and illustrated by the International Association for the Study of Pain (IASP). Here is our revised manuscript.*

*“In this network meta-analysis, we will include participants diagnosed with chronic cancer pain, according to the criteria defined by the International Association for the Study of Pain (IASP)(33). While it is generally accepted that chronic pain is defined as pain lasting longer than three months, the precise concept of time is not stressed in cancer pain because cancer patients may experience rapid disease progression and shortened survival times. Therefore, we will define chronic cancer pain is a kind of chronic pain caused by the primary cancer itself or metastases (chronic cancer pain) or its treatment (chronic postcancer treatment pain) (33).*

*We will include randomized controlled trials (RCTs) that feature a control group for efficacy comparison. The control group can receive a placebo, conventional treatment, or any intervention different from the primary treatment method. If a study lacks a control group, it must demonstrate that the target intervention has higher efficacy compared to other interventions applied in the sample.*

*The studies must employ a randomized controlled trial design to assess the effectiveness of both pharmacological and non-pharmacological treatments for chronic cancer pain patients. Non-pharmacological interventions must not impose any pharmacological effects on participants.*

*Participants must be adult males or females, at least 18 years old, experiencing pain caused by cancer. Both race and gender will be considered independent variables in the research.”*

**Comment 5: "The experimental group received medication analgesia or combination therapy (drug therapy along with non-drug therapy, conventional analgesics and unconventional analgesics); (3) The research type of literature is randomized controlled study; (4) The primary outcome measures are pain intensity score and total effective rate of treatment; (5) Secondary indicators are pain onset time, duration of relief, and quality of life score".**

**- This entire sentence should not be in the "Population" section. It's not the appropriate place.**

*Response:* Thank you for your great advice. *We removed these sentences, and separate them into different paragraphs. As for the research type which has been put in the study design section. We have set and defined the primary and secondary outcomes in different paragraphs.*

**Comment 6 : Exclusion criteria**

**- Much information is unnecessary, such as "Non-randomized controlled research, including reviews, case studies, and trials on animals". The PICOs Criterion should already be clear enough. Furthermore, this section should be the last one, after "Population", "Intervention", "Comparator", "Outcomes", and "Design".**

*Response: Yes, some criteria are not necessary. We followed your good advice. Here is the revised manuscript of exclusion criteria.*

*“Exclusion Criteria*

*We will exclude randomized controlled trials (RCTs) where participants' primary health condition is not chronic cancer pain or where the studies do not retain complete study data. Studies will be excluded if they do not meet the following criteria: they do not have a control group that received a different treatment from the one being studied; they do not demonstrate substantial efficacy of the target therapy compared to the other therapies included in the study; they include individuals who are awaiting a biopsy or a diagnosis; they conduct pain research specifically focused on diseases other than cancer; they have too broad inclusion criteria that include participants who do not meet the requirements of this study.”*

**Comment 7. Comparator**

**- Make it clear that for network meta-analysis, due to the very nature of this study, comparisons of interventions will also be compared with each other.**

*Response: Thank you, we followed your nice advice. In this network meta-analysis, comparisons of interventions will be made with each other.*

**Comment 8. Outcomes**

**- "Pain intensity was used as the primary outcome measure and measured using measurement tools". Why is the sentence in the past? This is a protocol. So, it must be in the future. Furthermore, it separates the outcomes as "primary(s)" and "secondary(s)".**

*Response:* *Yes, we realized that we made very a big mistake of our language, and we had revised and reviewed our language to make sure they are appropriate. We separate the primary and secondary outcomes into different paragraphs. Here is the revised outcomes.*

*“Outcomes*

*The results will be categorized into primary and secondary outcomes. We will include studies that assessed at least one of the following indicators:*

*Primary outcomes*

*Pain intensity*

*We will prioritize obtaining data from a single-dimensional pain scale. We will prioritize extracting numerical rating scale (NRS) data. If the studies included in the analysis do not utilize NRS, we will include the data in the following order: visual analog scale (VAS), verbal rating scale (VRS), verbal descriptor scale (VDS), Wong Baker faces pain scale revision (FPS-R), and Mankoski pain scale (MPS). The examination of multidimensional pain tools will only be considered if there is no alternative tool available for a single dimensional pain scale. We are going to use the multi-dimensional assessment tool in following orders if there is no single-dimensional pain scale being used in the included trials: brief pain inventory (BPI), short-form McGill pain questionnaire (SF/MPQ), and global pain scale (GPS). We will prioritize extracting the average pain, mildest pain, most severe pain, and pain data during rest and activity over the past 24 hours. The pain intensity assessment tool will be described in the trial's characteristics.*

*Total effective rate of treatment*

*If the study does not clearly disclose the total effective rate of treatment data, we will calculate them by hand. To calculate the total effective rate of treatment, we will divide the total number of effective treatments in the study by the total number of interventions. A effective treatment is defined as a reduction of at least 40% in patient pain intensity or frequency before and after treatment. The total effective rate of the treatment assessment tool will be described in the trial's characteristics.*

*Onset of analgesia*

*The onset of analgesia will be identified by assessing the time it takes for patients to experience substantial pain alleviation or complete elimination of pain following analgesic treatment.*

*Quality of life*

*We will prioritize collecting data from the European Organization for Research and Treatment of Cancer Quality of Life Questionnaire 30-Item Core Instrument (EORTC QLQ-C30). If the EORTC QLQ-C30 cannot be obtained from the studies included, we will collect data from the Functional Assessment of Cancer Therapy General Scale (FACT-G). We will also include other quality of life assessment tools, and quality of life assessment tool will be listed in the trial's characteristics as well.*

*Secondary outcome*

*Adverse reactions*

*As secondary outcome indicators, we will include treatment reactions such as nausea, vomiting, constipation, respiratory depression, fluctuations in blood pressure, and death. Since objective techniques cannot accurately measure subjective symptoms, we will exclude subjective symptom measurement instruments from the report. For measurable adverse reactions such as blood pressure, we will report them in the adverse reactions section.”*

**Comment 9: Study design**

**"We divide intervention measures into two categories based on their mechanisms: pharmacological intervention and non-pharmacological intervention, also include them in joint interventions. The databases PubMed, Web of Science, the Cochrane Library, MEDLINE, Embase, and CINAHL will all be searched. The list will only contain items that have been published in English; unpublished pieces will not be included. "Cancerous pain" and "randomized controlled trials" will be included in the search phrases".**

**- This entire sentence should be removed from this place. Please state here only which randomized controlled trials will be included. Crossover studies will be considered in their complete form or only if there is a washout period for a few weeks? Will there be a restriction on language or publication date?**

*Response: We removed the mentioned sentences, and there is no restriction on language or publication date. Here is our revised manuscript.*

*“We will carry out a systematic search of published randomized controlled trials (group, crossover, and parallel) in the PubMed, Web of Science, Cochrane Library, MEDLINE, Embase, and CINAHL databases, without language or date restrictions.*

*Study design incorporates parallel groups, cross-over, and cluster randomized controlled trials. In crossover designs, only data from the initial trial period will be retrieved to exclude any potential carryover effects. All included studies must have a minimum of two different intervention measures.”*

**Comment 10: After the PICOs criterion, create a section "Electronic search strategies", and include information about which databases will be searched, whether they will also search gray literature, whether the authors of articles with missing data will be contacted, and whether perform hand searches for potentially eligible articles.**

*Response: We added this part and here is our correction below:*

***“****Electronic search strategies*

*We will establish an electronic literature search strategy document, which will be implemented in PubMed, Web of Science, the Cochrane Library, MEDLINE, Embase, and CINAHL without any language or date restrictions. Additionally, we will conduct a thorough investigation of grey literature and make efforts to contact the authors of articles that lack necessary information. We will also manually search for published systematic reviews on this topic and include any relevant full-text articles from their reference lists.”*

**Comment 11: After the search section, create a "Search strategy" section, and describe the search strategies that will be used (provide as supplementary material). Describe the terms Medical Subject Headings (MeSH) and their synonyms and Boolean operators (when possible) to improve searches.**

*Response: We followed your suggestion, and created “Search strategy” section. Here is our correction below:*

***“****Study strategy*

*The search method was guided by the PICOS criteria and will consist of three sets of terms pertaining to (1) randomized trials, (2) chronic cancer pain, and (3) therapies. To enhance search results, we will utilize the Medical Subject Headings (MeSH), together with their synonyms and free-text keywords found in titles and abstracts.. Here are the MeSH terms that we are going to search in PubMed: Cancer Pain, Randomized Controlled Trial, Yoga, Music Therapy, etc. The detailed literature retrieval approach can be found in the S4 File.”*

**Comment 12: Create a section "Risk of bias across studies" and describe CINEMA. It shouldn't be in the section it is in.**

*Response:*

*Thank you for your suggestion. We have created a new section titled "Risk of Bias Across Studies" and moved the description of CINEMA to this section to ensure appropriate organization and clarity.*

**Comment 13: Strategy for data synthesis and analysis**

**（1）- Will heterogeneity be measured using the I² statistic? To estimate heterogeneity between studies, will the DerSimonian & Laird estimator be used?**

*Response:*

*Thank you for your comment. We have clarified that heterogeneity will be measured using the I² statistic. To estimate heterogeneity between studies, we will use the DerSimonian & Laird estimator.*

**（2）- Will a number of simulations and thin values be chosen by checking autocorrelation plots and trace plots?**

*Response:*

*Thank you for your comment. We have clarified that a number of simulations and thin values will not be chosen by checking autocorrelation plots and trace plots.*

**（3）- If it is a Bayesian network meta-analysis, will the results be presented with credible intervals through a league table?**

*Response:*

*Thank you for your comment. We have clarified that if it is a Bayesian network meta-analysis, the results will be presented with credible intervals through a league table.*

**（4）- "To assess the presence of publication bias or small sample effects, a corrective funnel plot was created for outcome measures having a literature volume of 10 or above." - PHRASE IN THE PAST?**

*Response:*

*Thank you for your comment. Here is our revised manuscript.*

*“We will use a calibrated funnel plot analysis to assess publication bias. If the funnel plot is asymmetrical, we will further investigate potential sources of publication bias.”*

**Comment 14: Discussion**

**（1）- "To compare the results and effects of various intervention measures, the ROB 2.0 tool was used to assess the risk of bias in the numerous studies that were included in the analysis(24)". - UNNECESSARY PHRASE.**

*Response: We followed your suggestion and removed this sentence.*

**（2）- Highlight in the Discussion that this protocol was written guided by the PRISMA-P statement, which will endorse the transparency, accuracy, and integrity of the study.**

*Response: We followed your suggestion. Here is our correction below:*

*“we will adhere to the PRISMA-P and PRISMA-NMA guidelines in our study composition.”*

**（3）- End the discussion with a sentence drawing attention to the lack of studies directly comparing different interventions in the scenario of cancer pain.**

*Response:* *Thank you for your comment. We have ended the discussion with the following sentence to highlight the gap in research and the purpose of our study:*

*"* *Currently, there is a lack of research explicitly comparing various chronic cancer pain interventions."*

We would like to express our great appreciation to you and reviewers for comments on our paper. Looking forward to hearing from you**.**

Thank you and best regards.

Yours sincerely,

Dr. Wang Yanru

No. 548 Binwen Road, Binjiang District, Hangzhou City, Zhejiang Province(310053)

Tel: +86-86613674
